# Depression and Psychological-Behavioral Responses Among the General Public in China During the Early Stages of the COVID-19 Pandemic: Survey Study

**DOI:** 10.2196/22227

**Published:** 2020-09-04

**Authors:** Weiyu Zhang, Xiaoting Yang, Jinfeng Zhao, Fengzhi Yang, Yajing Jia, Can Cui, Xiaoshi Yang

**Affiliations:** 1 Department of Social Medicine, School of Public Health China Medical University Shenyang China; 2 Department of Gastroenterology, Benxi General Hospital Liaoning Health Industry Group Benxi China; 3 The University of Auckland Auckland New Zealand

**Keywords:** depression, COVID-19, social support, the general public

## Abstract

**Background:**

The COVID-19 pandemic has recently spread dramatically worldwide, raising considerable concerns and resulting in detrimental effects on the psychological health of people who are vulnerable to the disease. Therefore, assessment of depression in members of the general public and their psychological and behavioral responses is essential for the maintenance of health.

**Objective:**

This study aimed to assess the prevalence of depression and the associated factors among the general public during the early stages of the COVID-19 pandemic in China.

**Methods:**

A cross-sectional survey with convenience sampling was conducted from February 11 to 16, 2020, in the early stages of the COVID-19 outbreak in China. A self-administrated smartphone questionnaire based on the Patient Health Questionnaire-9 (PHQ-9) and psychological and behavioral responses was distributed to the general public. Hierarchical multiple regression analysis and multivariate logistic regression analysis were conducted to explore the associated factors of depression.aA cross-sectional survey with convenience sampling was conducted from February 11 to 16, 2020, in the early stages of the COVID-19 outbreak in China. A self-administrated smartphone questionnaire based on the Patient Health Questionnaire-9 (PHQ-9) and psychological and behavioral responses was distributed to the general public. Hierarchical multiple regression analysis and multivariate logistic regression analysis were conducted to explore the associated factors of depression.

**Results:**

The prevalence of depression (PHQ-9 score ≥10) among the general public during the COVID-19 pandemic was 182/1342 (13.6%). Regression analysis indicated that feeling stressed, feeling helpless, persistently being worried even with support, never feeling clean after disinfecting, scrubbing hands and items repeatedly, hoarding food, medicine, or daily supplies, and being distracted from work or study were positively associated with depression, while social support and being calm were negatively associated with depression.

**Conclusions:**

The general public suffered from high levels of depression during the early stages of the COVID-19 pandemic. Thus, COVID-19–related mood management and social support should be provided to attenuate depression in the general public.

## Introduction

COVID-19 is a highly infectious disease that was identified at the end of 2019 and has been listed as Public Health Emergency of International Concern by the World Health Organization [1,2]. COVID-19 rapidly spread to 31 provinces, municipalities, and autonomous regions in China and then spread worldwide, developing into a serious global pandemic and resulting in harm to public health, the global economy, and social development. As of 4 PM CST, July 2, 2020, 10,726,907 confirmed cases and 514,458 deaths have been reported from 26 countries around the world; 85,264 confirmed cases and 4648 deaths have been reported in China [3,4].

The unexpected outbreak of COVID-19 resulted in adverse impacts on the general public, restricting normal activities and disrupting festive plans. A series of immediate emergency responses were initiated by Chinese government to prevent the spread of COVID-19. The responses included sealing off cities and provinces, restrictions on travel, control of civilian air traffic, self-quarantine, delaying the resumption of work and school, and setting up targeted hospitals to receive and treat patients. The rapid spread of COVID-19 and uncertainty related to the disease have created a great deal of stress and difficult predicaments for the public; this has resulted in the emergence of mental disorders, such as depression and anxiety [5]. Scarcities of medicine and personal protective equipment (eg, antibiotics, masks, alcohol sanitizer) and basic food supplies (eg, cereals and vegetables) during the earliest weeks of the pandemic seriously affected the mental health of the public [6,7]. Recent studies revealed that extensive exposure to COVID-19 pandemic stress can render the public vulnerable to depression [8].

The psychological and behavioral responses to the COVID-19 pandemic can be vulnerability factors of mental disorders during pandemics [9,10]. Previous research has indicated that adverse psychological and behavioral responses to public health emergencies may exacerbate the spread of disease and induce the development of mental disorders such as acute stress disorder, panic attacks, posttraumatic stress disorder, anxiety and depression, and even suicidal tendencies [11-17]. However, the patterns of psychological responses to pandemics are various and unpredictable [18], and they can have considerable influences on the psychological health of the public. A great number of previous studies indicate that negative emotionality, maladaptive cognitive style, and low levels of optimism are associated with the risks of negative psychological impacts [9,19,20]. People who overestimate threats or lack tolerance of uncertainty in pandemics can become highly worried and anxious during pandemics [21]. Perception of stress and helplessness arising from the stressful situations of the epidemic could have direct effects on the mental health of individuals and lead to prevalence of depression and anxiety [22,23]. Moreover, psychoneuroimmunology studies indicate that people with predisposition to emotional disorders may be particularly vulnerable as a result of immune responses [24,25]. However, positive emotions such as optimism play a moderating role in the relationship between stress and depression, and lower optimism is associated with affective disorders [26,27]. Additionally, it has been observed that during pandemics, people can share negative emotions, including disappointment, anger, stress, fear, and anxiety, in social life; this is also known as emotional contagion [28].

According to the cognitive-behavioral model of health anxiety, people who experience excessively high health anxiety are likely to become highly anxious during a pandemic and engage in maladaptive behaviors, such as excessive hand washing [9,18]. Studies of the concept of the behavioral immune system indicate that people can respond to pandemics excessively (ie, to medically unnecessary levels) and may avoid infection-related contaminants [9,29]. The information-motivation-behavior skill model and the modified behavioral framework mentioned in recent studies indicate that motivation, behavior skills and positive perception of risk have direct effects on health behaviors [30,31]. Conversely, to maintain health and avoid infection, the general public may also behave irrationally (eg, superstitious behaviors, overdose of vitamins, and use of herbal supplements or even folk remedies) when people are excessively worried or anxious during pandemics [32,33]. In a recent study, according to the conceptual model of Stimulus-Organism-Response, governance such as lockdown measures taken to cut off channels of infection decreased psychological distance, leading to a buffer effect on perceived risks and anxiety [34]. Adversity in pandemics, such as economic depression and shortage of supplements, can lead to antisocial behaviors such as violence, rioting, looting, and even civil unrest and mass panic [35]. Scientific prediction and appropriate guidance of public behaviors have important implications for people’s mental health [36,37].

Social support, which is perceived as care or help from others, has a protective effect on depression by regulating stress, as presented in previous studies [38,39], and it is closely associated with the generation, control, and prevention of mental disorders. Adequate social support and appropriate sources of support are beneficial to the public health because they can release stress, maintain individuals’ emotional responses, and prevent mental symptoms [40].

The COVID-19 pandemic has resulted in adverse effects on public mental health, which has recently raised considerable concerns [41,42]. Studies have been conducted to assess and prevent psychological health crises during the outbreak of COVID-19 [43]. As a result of the pandemic, the public has reported discomforts such as heavy mental health burdens, poor sleep quality, and psychological distress [44-48]. Therefore, in our study, we aimed to appraise depression and explore its associated factors, including psychological responses, behavioral responses, and social support, among the general public during the early stages of the COVID-19 pandemic. Our findings can be used to provide evidence-based advice on early psychological and behavioral interventions to reduce depression in the general public.

## Methods

### Participants, Procedure, and Ethics Statement

A cross-sectional survey with convenience sampling was conducted in mainland China from February 11 to 16, 2020. A web link and quick response code were distributed via WeChat, a widely used social network platform in China, to collect self-administrated questionnaires with support from the Environmental Health Institute at China Medical University. Participants anonymously completed a questionnaire in 15 to 20 minutes comprising questions based on the Patient Health Questionnaire-9 (PHQ-9) and concerning psychological and behavioral responses to the COVID-19 pandemic. Participants included in this study met the following inclusion criteria: aged ≥18 years; able to read and write Chinese; able to use WeChat to complete the questionnaire independently; willing to participate and provide signed web-based informed consent. Any participant meeting one or more of the following criteria was excluded: receiving treatment for any psychological illness; having significant visual impairment; having a history of drug dependence; having been diagnosed with a disease that would prevent them from completing the questionnaire independently.

This survey recruited a total of 1675 adults from the general public in 3 municipalities and 22 provinces or autonomous regions in China. Each participant was well informed of the aims, funding, and contents of the questionnaire and the commitment to the privacy of the participants. Questionnaires that were answered completely and logically were regarded as valid. A total of 1342/1675 participants provided valid answers to the questionnaire, resulting in a valid response rate of 80.12%. This study conformed to ethical standards and was conducted in accordance with the Helsinki Declaration as revised in 1989. The Ethics Committee of China Medical University approved the protocols of this study.

### Demographic Characteristics of the Participants

The demographic characteristics of the participants that were collected included gender (male, female), age (≤35 years, >35 years), marital status (married, other), occupation, education, and monthly income. Age was categorized according to proportionality, population distribution, and previous studies on the relationship between age and use of WeChat [49,50]. Occupation was categorized as government worker/civil servant/village committee worker/enterprise employee, health care worker, student, teacher/lawyer/journalist, and other. Education was categorized as below junior college, junior college, and bachelor’s degree and above. Monthly income (RMB) was classified as ≤¥5000 (≤US $725.19), ¥5001 to ¥10,000 (US $725.34 to $1,450.39), and >¥10,000 (>US $1,450.39).

### Measurement of Depression

Depression was assessed using the 9-item PHQ-9, which is one of the most widely used tools to assess depression [51]. Each item asked about the situation in the past two weeks using a 4-point Likert-type scale with choices of “not at all,” “a few days,” “more than half of the two weeks,” and “nearly every day,” giving a total score between 0 and 21. A score above 10 is regarded to be indicative of depression [52-55]. The Cronbach α coefficient of the PHQ-9 in this study was .91.

### Measurement of Psychological Responses

Psychological responses were measured by self-developed questions. Psychological responses included “feeling stressed,” “feeling helpless,” “persistently being worried even with support,” “being calm,” and “being optimistic.” Answers were grouped into three categories of “disagree,” “not sure,” and “agree” to assess the participants’ psychological responses during the past two weeks.

### Measurement of Behavioral Responses

Behavioral responses were measured by self-developed questions. Options for reflecting behavioral responses for this study included “never feeling clean after disinfecting; scrubbing hands and items repeatedly” (answers were “no,” “sometimes,” and “always”), “hoarding food, medicine, or daily supplies,” “social avoidance to avoid infection,” and “being distracted from work or study.” Answers were grouped into 3 categories of “disagree,” “not sure,” and “agree” to evaluate the participants’ behavioral responses during the past two weeks.

### Measurement of Social Support

Social support was assessed by a “yes or no” question that asked whether people had received social support in the past two weeks.

### Statistical Analyses

All analyses were performed using SPSS version 23.0 for Windows (IBM Corporation). A two-tailed probability value <.05 was considered statistically significant. We used *t* tests and one-way analysis of variance to compare differences in depression among categorical variables. Hierarchical multiple regression (HMR) analysis was conducted to test incremental variance using the following independent variables: Step 1: demographic characteristics of the general public; Step 2: psychological responses to the COVID-19 pandemic; Step 3: behavioral responses to the COVID-19 pandemic; and Step 4: social support. The depression scores were continuous in HMR and used as the dependent variables. Standardized parameter estimates (standardized β) were used to compare the magnitude of associations of the independent variables. The depression scores in the multivariate logistic regression analysis were binary (depression or no depression) with a cutoff score of 10. Multivariate logistic regression analysis was performed to explore risk factors associated with depression using odds ratios (ORs). Among all categorically independent variables, items for which more than 95% of individuals had the same response were not included in the data analysis. A two-tailed *P* value <.05 was considered to be statistically significant.

## Results

### Demographic Characteristics and Depression Distribution of the Participants

A total of 1342 subjects participated in this study, giving a valid response rate of 1342/1675 (80.12%). The distribution of the demographic characteristics of the participants, prevalence of depression, and univariate analysis of depression are listed in Table 1. The prevalence of depression among the general public was 182/1342 (13.60%).

The 1342 participants included 500 men (37.26%) and 842 women (62.74%). Approximately one-third of the participants (467/1342, 35.77%) had a bachelor’s degree or higher degree. Depression scores for the participants who were aged <35 years were significantly higher than those of the other participants (*P*=.001). Participants who were married tended to have lower depression scores than participants who were not (*P*<.001). Government workers, civil servants, village committee workers, enterprise employees, and health care workers had lower depression scores than those engaging in other occupations (*P*<.001).

**Table 1:** Characteristics of the general public and distribution of depression in China during the early stages of the COVID-19 pandemic (N=1342).

Variable				Total, n (%)		Depression, n (%)		No depression, n (%)		Depression score, mean (SD)		*P* value
**Demographic characteristics**												
	**Gender**											.30
		Male	500 (37.26)		66 (13.20)		434 (86.80)		4.04 (5.68)			
		Female	842 (62.74)		116 (13.78)		726 (86.22)		4.35 (5.13)			
	**Age (years)**											.001
		≤35	597 (44.49)		98 (16.42)		499 (83.58)		4.80 (5.57)^a^			
		>35	745 (55.51)		84 (11.28)		661 (88.72)		3.78 (5.11)			
	**Marital status**											<.001
		Married	898 (66.92)		107 (11.92)		791 (88.08)		3.85 (5.07)			
		Other	444 (33.09)		75 (16.89)		369 (83.11)		5.03 (5.77)^a^			
	**Occupation**											<.001
		Government worker, civil servant, village committee worker, or enterprise employee	377 (28.09)		34 (9.02)		343 (90.98)		3.56 (4.99)			
		Health care worker	279 (20.79)		30 (10.75)		249 (89.25)		3.59 (4.71)			
		Student	203 (15.13)		37 (18.23)		166 (81.77)		5.00 (5.29)^a^			
		Teacher, lawyer, or journalist	244 (18.18)		37 (15.16)		207 (84.84)		4.48 (5.36)^b^			
		Other	239 (17.81)		44 (18.41)		195 (81.59)		5.19 (6.30)^a^			
	**Education**											.08
		Below junior college	166 (12.37)		165 (15.15)		141 (84.85)		4.41 (5.74)			
		Junior college	709 (52.83)		107 (15.09)		602 (84.91)		4.49 (5.67)^b^			
		Bachelor’s degree and above	467 (34.80)		50 (10.71)		417 (89.29)		3.80 (4.62)			
	**Monthly income (¥)^c^**											.006
		≤5000	445 (33.16)		77 (17.30)		368 (82.70)		3.72 (4.98)			
		5001-10,000	535 (39.87)		53 (9.91)		482 (90.09)		3.21 (4.17)			
		>10,000	362 (26.97)		52 (14.36)		310 (85.64)		3.54 (4.44)			
**Psychological Responses**												
	**Feeling stressed**											<.001
		Disagree	370 (27.57)		16 (4.32)		354 (95.68)		1.72 (3.48)			
		Not sure	634 (47.24)		63 (9.94)		571 (90.06)		3.85 (4.48)^a^			
		Agree	338 (25.19)		103 (30.47)		235 (69.53)		7.74 (6.60)^a^			
	**Feeling helpless**											<.001
		Disagree	554 (41.28)		103 (18.59)		451 (81.41)		2.44 (4.18)			
		Not sure	443 (33.01)		49 (11.06)		394 (88.94)		4.09 (4.61)^a^			
		Agree	345 (25.71)		30 (8.70)		315 (91.30)		7.32 (6.41)^a^			
	**Persistently being worried even with support**											<.001
		Disagree	648 (48.29)		24 (3.70)		624 (96.30)		2.05 (3.46)			
		Not sure	313 (23.32)		37 (11.82)		276 (88.18)		4.30 (4.67)^a^			
		Agree	381 (28.39)		121 (31.76)		260 (68.24)		7.91 (6.39)^a^			
	**Being calm**											<.001
		Agree	521 (38.82)		33 (6.33)		488 (93.67)		2.74 (4.44)			
		Not sure	548 (40.83)		73 (13.32)		475 (86.68)		4.36 (4.94)^a^			
		Disagree	273 (20.35)		76 (27.84)		197 (72.16)		6.86 (6.53)^a^			
	**Being optimistic**											<.001
		Agree	410 (30.55)		39 (9.51)		371 (90.49)		3.32 (5.10)			
		Not sure	425 (31.67)		42 (9.88)		383 (90.12)		3.63 (4.50)			
		Disagree	507 (37.78)		101 (19.92)		406 (80.08)		5.50 (5.99)^a^			
**Behavioral responses**												
	**Never feeling clean after disinfecting; scrubbing hands and items repeatedly**											<.001
		No	543 (40.46)		35 (6.45)		508 (93.55)		2.73 (4.41)			
		Sometimes	402 (29.96)		60 (14.93)		342 (85.07)		4.83 (4.96)^a^			
		Always	397 (29.58)		87 (21.91)		310 (78.09)		5.71 (6.28)^a^			
	**Hoarding food, medicine, or daily supplies**											<.001
		Disagree	595 (44.34)		55 (9.24)		540 (90.76)		3.07 (4.60)			
		Not sure	492 (36.66)		57 (11.59)		435 (88.41)		4.27 (4.66)^a^			
		Agree	255 (19.00)		70 (27.45)		185 (72.55)		6.89 (7.00)^a^			
	**Social avoidance to avoid infection**											.053
		Disagree	275 (20.49)		36 (13.09）		239 (86.91)		3.89 (5.24)			
		Not sure	547 (40.76)		63 (11.52)		484 (88.48)		3.99 (4.76)			
		Agree	520 (38.75)		83 (15.96)		437 (84.04)		4.68 (5.93)			
	**Being distracted from work or study**											<.001
		Disagree	443 (33.01)		3 (0.68)		440 (99.32)		1.23 (2.33)			
		Not sure	379 (28.24)		32 (8.44)		347 (91.56)		3.27 (3.90)^a^			
		Agree	520 (38.75)		147 (28.27)		373 (71.73)		7.51 (6.24)^a^			
	**Social support**											**<.**001
		Yes	1198 (89.27)		146 (12.19)		1052 (87.81)		4.00 (5.08)			
		No	144 (10.73)		36 (25.00)		108 (75.00)		6.21 (6.89)^a^			

^a^Significant at the 0.01 level (two-tailed).

^b^Significant at the 0.05 level (two-tailed).

^c^1 ¥ = US $0.15.

### Psychological and Behavioral Responses to the COVID-19 Pandemic

The distribution of depression scores according to psychological and behavioral responses to the COVID-19 pandemic are shown in Table 1. The participants who disagreed that they felt stressed or helpless tended to have lower depression scores than those who agreed or were not sure (*P*<.001). Depression scores were lower among participants who were persistently worried even with support (*P*<.001). The depression scores for the individuals who agreed that they were calm or optimistic about the COVID-19 pandemic were significantly lower (*P*<.001).

The participants who never felt clean after disinfecting and who scrubbed their hands and items repeatedly had significantly higher depression scores (*P*<.001). Participants who agreed that they hoarded food, medicine, or daily supplies tended to have significantly higher depression scores (*P*<.001). Scores were higher for the participants who agreed that they were distracted from work or study (*P*<.001).

The results also showed that participants who reported that they did not receive any support from others when they had difficulties during the COVID-19 pandemic had a significantly higher score of depression than those who received social support (*P*<.001).

### Risk Factors of Depression

**Figure 1:**
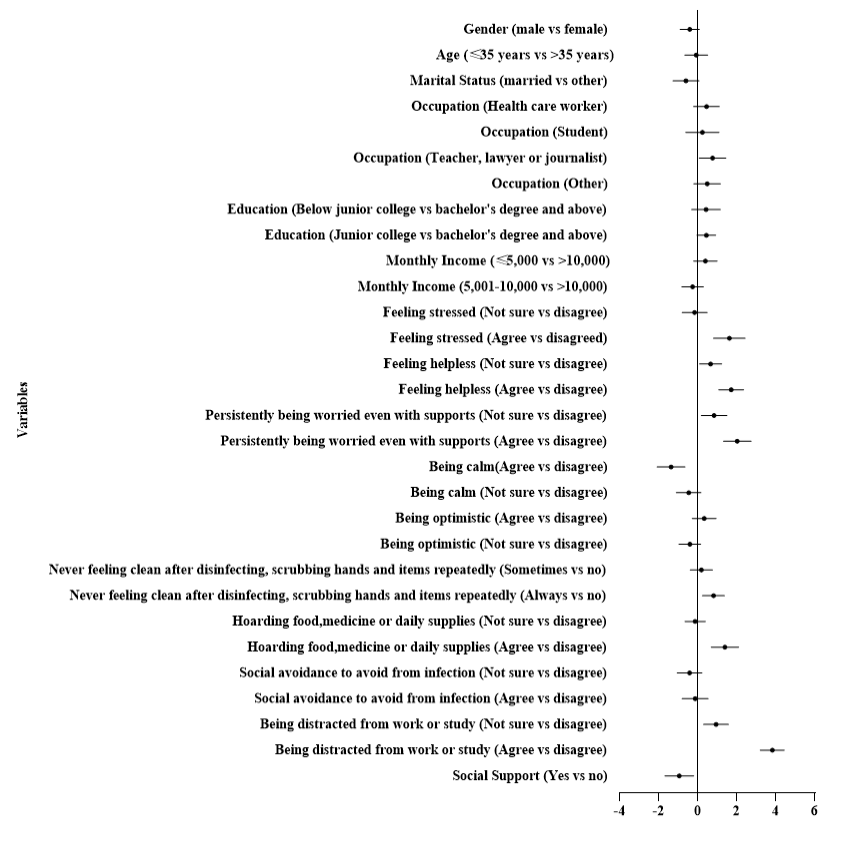
Forest plot of the risk factors of depression (hierarchical multiple regression).

Table 2 shows the final results of the HMR models of depression among the general public. A total of 42.1% of the variance was explained by the final model. The *R*^2^ changes indicated that the incremental variances explained by each block of variables were 3.1%, 28.6%, 10.1%, and 0.3% for demographic characteristics, psychological responses, behavioral responses, and social support, respectively. In the final model of the HMR and the forest plot (Figure 1), the risk factors of depression included feeling stressed; feeling helpless; persistently being worried, even with support; never feeling clean after disinfecting, and scrubbing hands and items repeatedly; hoarding food, medicine, or daily supplies; and being distracted from work or study (all *P*<.01). However, being calm and social support were negatively associated with depression.

**Table 2:** Hierarchical linear regression analysis of depression among the general population in China during the early stages of the COVID-19 pandemic.

Variable			β	Standardized β	95% CI	*P* value	Adjusted *R*^2^	Δ*R*^2^
**Demographic characteristics**							0.023	0.031
	Gender (male vs female)		–0.429	–0.039	–0.926 to 0.068	.09		
	Age (>35 years vs ≤35 years)		–0.082	–0.008	–0.675 to 0.511	.79		
	Marital status (married vs other)		–0.669	–0.059		.06		
	**Occupation**							
		Health care worker	0.407	0.031	–0.259 to 1.073	.23		
		Student	0.277	0.019	–0.599 to 1.154	.54		
		Teacher, lawyer, or journalist	0.745	0.054	0.066 to 1.425	.03^a^		
		Other	0.502	0.036	–0.188 to 1.192	.15		
	**Education**							
		Below junior college vs bachelor’s degree and above	0.437	0.027	–0.299 to 1.174	.25		
		Junior college vs bachelor’s degree and above	0.433	0.040	–0.053 to 0.919	.08		
	**Monthly income (¥)^b^**							
		≤5000 vs >10,000	0.382	0.034	–0.226 to 0.991	.22		
		5001-10,000 vs >10,000	–0.231	–0.021	–0.791 to 0.330	.42		
**Psychological responses**							0.306	0.286
	**Feeling stressed**							
		Not sure vs disagree	–0.161	–0.015	–0.805 to 0.482	.62		
		Agree vs disagree	1.631	0.133	0.813 to 2.448	<.001^c^		
	**Feeling helpless**							
		Not sure vs disagree	0.665	0.059	0.097 to 1.234	.02^a^		
		Agree vs disagree	1.717	0.141	1.076 to 2.358	<.001^c^		
	**Persistently being worried even with support**							
		Not sure vs disagree	0.822	0.065	0.156 to 1.488	.016^a^		
		Agree vs disagree	2.016	0.170	1.299 to 2.733	<.001^c^		
	**Being calm**							
		Agree vs disagree	–1.365	–0.125	–2.081 to –0.649	<.001^c^		
		Not sure vs disagree	–0.477	–0.044	–1.115 to 0.160	.14		
	**Being optimistic**							
		Agree vs disagree	0.318	0.027	–0.296 to 0.933	.31		
		Not sure vs disagree	–0.407	–0.035	–0.968 to 0.153	.15		
**Behavioral responses**							0.404	0.101
	**Never feeling clean after disinfecting; scrubbing hands and items repeatedly**							
		Sometimes vs no	0.175	0.015	–0.398 to 0.748	.55		
		Always vs no	0.803	0.069	0.231 to 1.374	.006^c^		
	**Hoarding food, medicine, or daily supplies**							
		Not sure vs disagree	–0.157	–0.014	–0.683 to 0.369	.56		
		Agree vs disagree	1.393	0.102	0.686 to 2.099	<.001^c^		
	**Social avoidance to avoid infection**							
		Not sure vs disagree	–0.421	–0.039	–1.060 to 0.218	.20		
		Agreed vs disagree	–0.121	–0.011	–0.793 to 0.550	.72		
	**Being distracted from work or study**							
		Not sure vs disagree	0.951	0.080	0.311 to 1.590	.004^c^		
		Agree vs disagree	3.842	0.351	3.219 to 4.464	<.001^c^		
Social support (yes vs no)			–0.943	–0.055	–1.682 to –0.205	.012^a^	0.406	0.003

^a^Significant at the .05 level (two-tailed).

^b^1 ¥ = US $0.15.

^c^Significant at the .01 level (two-tailed).

**Figure 2:**
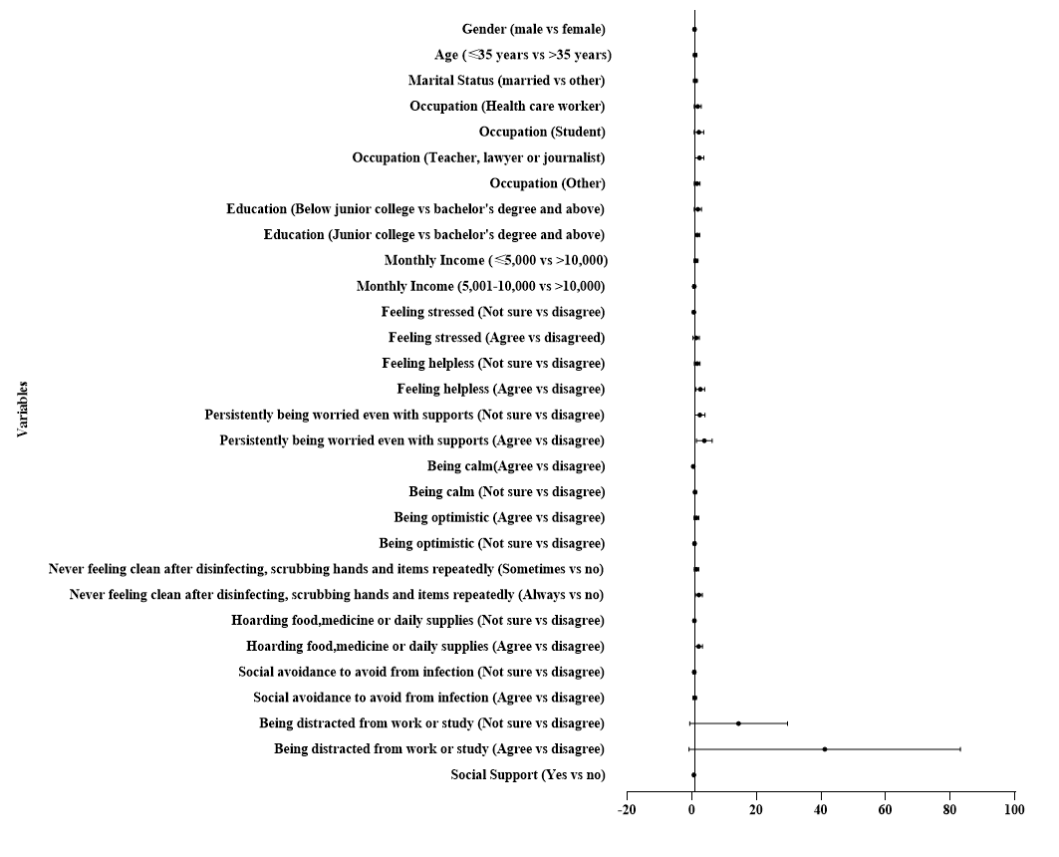
Forest plot of the risk factors of depression (multivariate logistic regression).

Table 3 presents the results of the multivariate logistic regression analysis. Feeling helpless (OR 2.341, 95% CI 1.367-4.009), persistently being worried even with support (OR 3.315, 95% CI 1.696-6.479), never feeling clean after disinfecting and scrubbing hands and items repeatedly (OR 1.941, 95% CI 1.153-3.267), hoarding food, medicine, or daily supplies (OR 1.822, 95% CI 1.012-3.279), and being distracted from work or study (OR 27.225, 95% CI 8.243-89.918) increased the risk of depression. However, being calm (OR 0.344, 95% CI 0.186-0.635) and social support (OR 0.529, 95% CI 0.308-0.908) decreased the chance of suffering from depression. Additionally, compared with other occupations, teachers, lawyers, and journalists (OR 2.053, 95% CI 1.117-3.776) had higher chances of depression. In the final model of the multivariate logistic regression and the forest plot (Figure 2), the risk factors of depression included occupation of teacher, lawyer, or journalist; junior college education; feeling helpless; persistently being worried, even with support; never feeling clean after disinfecting and scrubbing hands and items repeatedly; hoarding food, medicine, or daily supplies; and being distracted from work or study (all *P*<.01). However, being calm and social support were protective factors of depression.

**Table 3:** Multivariate logistic regression analysis exploring factors of depression among the general population in China during the early stages of the COVID-19 pandemic.

Variable			Odds ratio	95% CI
**Demographic characteristics**				
	Gender (male vs female)		0.742	0.478-1.150
	Age (>35 years vs ≤35 years)		0.885	0.532-1.474
	Marital status (married vs other)		0.936	0.531-1.651
	**Occupation**			
		Health care worker	1.530	0.804-2.914
		Student	1.810	0.872-3.754
		Teacher, lawyer, or journalist	2.049	1.115-3.768
		Other	1.403	0.765-2.573
	**Education**			
		Below junior college vs bachelor’s degree and above	1.596	0.839-3.035
		Junior college vs bachelor’s degree and above	1.572	1.018-2.428
	**Monthly income (¥)^a^**			
		≤5000 vs >10,000	1.160	0.697-1.931
		5001-10,000 vs >10,000	0.617	0.374-1.018
**Psychological responses**				
	**Feeling stressed**			
		Not sure vs disagree	0.465	0.216-1.003
		Agree vs disagree	1.018	0.436-2.376
	**Feeling helpless**			
		Not sure vs disagree	1.455	0.826-2.562
		Agree vs disagree	2.348	1.373-4.016
	**Persistently being worried even with support**			
		Not sure vs disagree	2.120	1.081-4.160
		Agree vs disagree	3.303	1.696-6.433
	**Being calm**			
		Agree vs disagree	0.345	0.188-0.636
		Not sure vs disagree	0.876	0.553-1.386
	**Being optimistic**			
		Agree vs disagree	1.262	0.729-2.186
		Not sure vs disagree	0.713	0.443-1.149
**Behavioral responses**				
	**Never feeling clean after disinfecting; scrubbing hands and items repeatedly**			
		Sometimes vs no	1.265	0.746-2.145
		Always vs no	1.942	1.158-3.257
	**Hoarding food, medicine, or daily supplies**			
		Not sure vs disagree	0.655	0.397-1.082
		Agree vs disagree	1.862	1.039-3.338
	**Social avoidance to avoid infection**			
		Not sure vs disagree	0.619	0.347-1.104
		Agree vs disagree	0.746	0.404-1.379
	**Being distracted from work or study**			
		Not sure vs disagree	9.131	2.648-31.495
		Agree vs disagree	26.810	8.128-88.430
Social support (yes vs no)			0.524	0.305-0.901

^a^1 ¥ = US $0.15.

## Discussion

### Principal Findings

This study revealed that 13.6% of the general public suffered from depression during the early stages of the pandemic of COVID-19 in China. The prevalence of depression was slightly lower than the prevalence before the outbreak of COVID-19 from previous studies [56-58] (13.8% vs 18.8%). The results also indicated that the prevalence of depression during the COVID-19 pandemic is almost identical to that during the SARS epidemic (11.9% vs 18.0%) among the general public in China [59,60]. Most of the research on mental disorders during public health emergencies in China focuses on the psychological status of patients or health care workers; depression in the general public is neglected and untreated [61-64]. The general public are extremely vulnerable to symptoms of depression during the COVID-19 pandemic. People with mental symptoms of depression should be identified and managed in a timely manner to improve their psychological health status [65].

Depression is closely associated with the psychological and behavioral responses of the general public during the COVID-19 pandemic. This study found that psychological responses contributed the most (28.6%) to the variance of depression. Feeling stressed, feeling helpless, and feeling persistently worried even with support were positively associated with depression, which is in agreement with previous studies indicating that the increase of perceived susceptibility to the epidemic is associated with the prevalence of mental disorders [66]. Perception of the risks of infection is a possible reason for negative psychological responses to pandemics and is highly correlated with distress in individuals [67-69]. Additionally, poor self-rated health status can be causative and can result in psychological burdens and excessive worries about the pandemic [70,71]. In addition, individuals who are worried even when they have support may be more prone to behavior problems, which can exacerbate the symptoms of depression [72,73].

Notably, being calm and optimistic are negatively associated with depression. Calmness and optimism can help people cope with problems rationally and may mediate the negative effects of perception of epidemic-related stress and indirectly attenuate the symptoms of depression [74]. On the other hand, positive cognition of public health emergencies has close correlations with stress and social functions [75]. Additionally, positive psychological capabilities such as calmness and optimism can ameliorate depression, in particular for people who experience higher levels of helplessness [76]. Lessened psychological capabilities can result in high susceptibility to anxiety and depression during negative emotional experiences or periods of adversity such as the COVID-19 pandemic. Thus, psychological management and regulation of the responses to a public health emergency may benefit from consciously controlling stress, helplessness, and worry and from maintaining calm and optimism.

This study showed that depression was significantly linked to behavioral responses. More than half of the participants (799/1342, 59.54%) reported that they never felt clean after they disinfected and scrubbed hands and items repeatedly, and 1067/1342 (79.51%) of these members of the general public practiced social avoidance to avoid the infection of COVID-19. Preventive behaviors are key to reducing the spread and impact of a pandemic. However, excessively preventive and avoidant behavioral responses, which possibly resulted from the perceived stress induced by the COVID-19 pandemic, were positively associated with depression. Recent studies have reported that psychological impacts, including depression, anxiety, and stress, are linked with the adoption of precautionary measures to prevent the spread of COVID-19 [16]. For example, using medical preventive equipment and washing after touching contaminated surfaces can provide potential psychological benefits by offering a sense of security and comport for the general public and can prevent the spread of COVID-19. However, these precautionary measures can also have negative effects on individuals’ mental health when they become excessive [44,77]. People may feel more susceptible to becoming infected during pandemics. Negative psychological impacts revealed by these behaviors can be easily popularized among the general public. This finding is consistent with other studies showing that protective behaviors such as avoiding sharing of utensils during meals and washing hands frequently increase the likelihood of stress, anxiety, and depression [78]. Health professionals should pay more attention to these behaviors, which can have adverse impacts on mental health, with a view to realizing early warning signs and conducting psychological interventions to protect vulnerable groups [79].

Specifically, social support appeared to be a protective factor for depression. Social support can enhance self-esteem and positive adaptation to stress and combat the detrimental effects of stress or worry on the development of depression [44]. Social support such as positive communications and entertainment may help individuals to recover quickly the from the COVID-19 outbreak [80]. In contrast, lack of social support can result in hostility and uncertainty in social life, thus increasing susceptibility to serious mental disorders, particularly depression [16]. Therefore, adequate and appropriate social support should be provided to prevent the development of mental disorders in the general public.

### Limitations

Several limitations should be acknowledged when presenting our findings. First, this survey was conducted with convenience sampling, which limits our ability to generalize the results of this study to the overall population. Second, this study was based on a self-administrated questionnaire completed via smartphone, which limited the diversity of the sample and the authenticity of the answers. Depression and the associated factors revealed in this study were limited to the early stages of the COVID-19 pandemic.

### Conclusion

The general public in China suffered from high levels of depression during the early stages of the COVID-19 pandemic. Being stressed, feeling helpless or worried, and being distracted from work or study increased the risks of depression. People who experience these adverse psychological responses should be provided with psychological interventions to reduce the symptoms of depression. Moreover, positive emotions and psychological responses such as calmness and optimism decreased the risks of depression and should be encouraged by health professionals and educators. Behavioral responses, including repeated cleaning, hoarding supplies, and distraction from work or study, were not only influencing factors of depression but also expressions of depression. Therefore, early detection and psychological and behavioral interventions should be developed to help people cope rationally with pandemic-related stress and improve the mental health of the general public.

## Abbreviations

HMR: hierarchical multiple regression
OR: odds ratio
PHQ-9: Patient Health Questionnaire-9
